# Automated whole-heart volumetrics and haemodynamics from 4D flow CMR magnitude images: development and validation of a deep learning model

**DOI:** 10.1093/ehjimp/qyag125

**Published:** 2026-07-09

**Authors:** Alexander Gall, Ciaran Grafton-Clarke, Rui Li, Zia Mehmood, Bahman Kasmai, Marina Hughes, Peter P Swoboda, Rob J van der Geest, Vassilios Vassiliou, Gareth Matthews, Pankaj Garg

**Affiliations:** Norwich Medical School, University of East Anglia, Norwich Research Park, Norwich NR4 7UQ, UK; Department of Cardiology, Norfolk and Norwich University Teaching Hospitals, Norwich NR4 7UY, UK; Norwich Medical School, University of East Anglia, Norwich Research Park, Norwich NR4 7UQ, UK; Department of Cardiology, Norfolk and Norwich University Teaching Hospitals, Norwich NR4 7UY, UK; Norwich Medical School, University of East Anglia, Norwich Research Park, Norwich NR4 7UQ, UK; Department of Cardiology, Norfolk and Norwich University Teaching Hospitals, Norwich NR4 7UY, UK; Norwich Medical School, University of East Anglia, Norwich Research Park, Norwich NR4 7UQ, UK; Department of Cardiology, Norfolk and Norwich University Teaching Hospitals, Norwich NR4 7UY, UK; Norwich Medical School, University of East Anglia, Norwich Research Park, Norwich NR4 7UQ, UK; Department of Cardiology, Norfolk and Norwich University Teaching Hospitals, Norwich NR4 7UY, UK; Department of Cardiology, Norfolk and Norwich University Teaching Hospitals, Norwich NR4 7UY, UK; Leeds Institute of Cardiovascular and Metabolic Medicine, University of Leeds, Leeds, UK; Division of Image Processing, Department of Radiology, Leiden University Medical Centre, Leiden, The Netherlands; Norwich Medical School, University of East Anglia, Norwich Research Park, Norwich NR4 7UQ, UK; Department of Cardiology, Norfolk and Norwich University Teaching Hospitals, Norwich NR4 7UY, UK; Norwich Medical School, University of East Anglia, Norwich Research Park, Norwich NR4 7UQ, UK; Department of Cardiology, Norfolk and Norwich University Teaching Hospitals, Norwich NR4 7UY, UK; Norwich Medical School, University of East Anglia, Norwich Research Park, Norwich NR4 7UQ, UK; Department of Cardiology, Norfolk and Norwich University Teaching Hospitals, Norwich NR4 7UY, UK

**Keywords:** artificial intelligence, deep learning, 4D flow, cardiac magnetic resonance, volumetrics, kinetic energy, energy loss

## Abstract

**Aims:**

4D flow cardiovascular magnetic resonance (CMR) offers a comprehensive haemodynamic assessment but is often limited by long acquisition times and complex post-processing. The magnitude images derived from 4D flow sequences contain time-resolved 3D anatomical information. We aimed to validate the anatomical accuracy of these images against standard cine imaging and develop an artificial intelligence (AI) model for automated segmentation to facilitate analysis.

**Methods and results:**

Forty patients prospectively identified from the PREFER-CMR registry underwent CMR, including standard cine stacks and 4D flow. The study consisted of two stages. In Stage 1, manual segmentation of the cardiac chambers and great vessels was performed on 4D flow magnitude images. These were validated against standard cine volumetrics (LV/RV) and normative reference values (LA/RA). In Stage 2, a fully automated deep learning algorithm was trained and validated. Advanced haemodynamic metrics were derived using both manual and AI segmentations to assess agreement. The study cohort (*n* = 40) had a mean age of 69.0 ± 17.2 years, and 60.0% were male. In Stage 1, 4D flow magnitude analysis demonstrated excellent correlations with cine measurements for LV end-diastolic volume (ρ = 0.98, ICC = 0.99) and RV end-diastolic volume (ρ = 0.97, ICC = 0.98). In Stage 2, the AI model achieved excellent segmentation performance (mean Dice similarity coefficient 0.88). Comparisons of haemodynamic metrics derived from AI vs. manual contours showed strong agreement (*r* ≥ 0.88 for all peak metrics).

**Conclusion:**

4D flow magnitude imaging provides accurate volumetrics. Deep learning automation of this process is feasible, allowing for rapid, comprehensive assessment of cardiac structure, function, and advanced energetics.

## Graphical Abstract

**Graphical Abstract:**
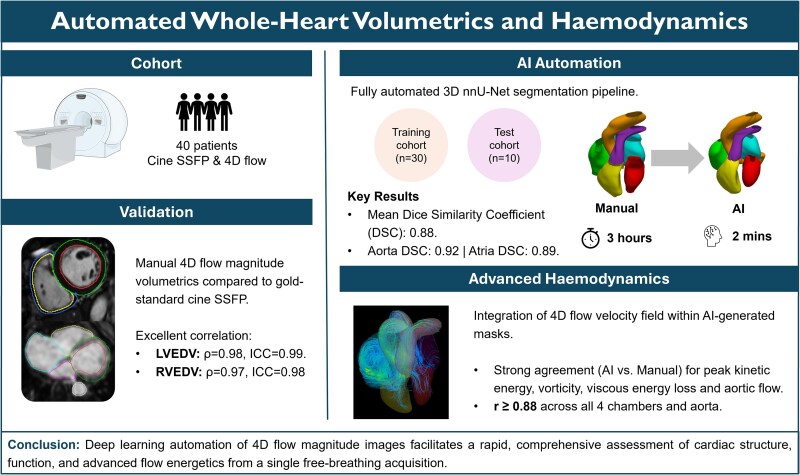

## Introduction

Cardiovascular magnetic resonance (CMR) is the reference standard for assessing cardiac morphology and function.^1^ Routine protocols typically rely on multiple 2D balanced steady-state free precession (SSFP) cine stacks to quantify ventricular volumes. This approach requires repeated breath-holds, which can be fatiguing for patients, time-consuming for radiographers and prone to misalignment artefacts.^2^

4D flow CMR has emerged as a powerful tool for visualizing complex haemodynamics, allowing for retrospective quantification of flow through any plane in the acquired volume.^3^ Beyond standard flow quantification, 4D flow enables the assessment of advanced energetic and fluid dynamic markers, including kinetic energy (KE), vorticity, and viscous energy loss (EL).^4–6^ These parameters offer deeper insights into cardiac efficiency, ventricular workload, and flow disturbances applicable across all cardiac diseases affecting both the left and right heart.^5–7^ However, the calculation of these metrics requires precise, time-resolved volumetric segmentation of the cardiac chambers to mask the velocity data correctly in a perfectly co-registered dataset.^3,8^

Traditionally, 4D flow is viewed as an ‘add-on’ sequence focused solely on velocity encoding. Crucially, however, the magnitude data acquired during 4D flow sequences provides time-resolved 3D anatomical information inherently co-registered with the velocity data.^9^ Recent work has demonstrated that these magnitude images can be used for accurate volumetric assessment, even in native (non-contrast) acquisitions.^10^ If accurate volumetric analysis can be performed directly on these magnitude images, it presents an opportunity to streamline CMR protocols.^9^ Furthermore, manual segmentation of 4D datasets is notoriously labour-intensive due to the large number of slices and phases, making automated solutions essential for clinical translation.^8^ While AI solutions exist for short-axis cine stacks, automated analysis of 4D flow magnitude anatomy remains underexplored, although recent deep learning approaches have shown promise in segmenting contrast-enhanced 4D flow datasets.^8,11,12^

This study was designed in two stages. In stage 1, we aimed to validate manual volumetric analysis of 4D flow magnitude images against the gold standard (cine SSFP) for ventricular volumes and normative references for atrial volumes.^13–15^ In stage 2, we first sought to develop and validate a deep learning model (convolutional neural network) to automate this segmentation. We then aimed to validate the utility of this AI model for deriving advanced haemodynamic indices (KE, Vorticity, and EL) by comparing AI-derived values against those obtained from manual segmentation.

## Methods

### Study population

We identified 40 patients from the prospective, Wellcome Trust funded PREFER-CMR registry at Norfolk and Norwich University Hospital. All patients underwent CMR with high quality 4D flow. Indications for scanning included valvular heart disease and heart failure (*Table 1*).

**Table 1:** Baseline characteristics of the study population (*n* = 40)

Demographics	All (*n* = 40)
Age (years)	69.0 ± 17.2
Sex (male), *n* (%)	24 (60.0%)
Height (cm)	169.3 ± 10.7
Weight (kg)	79.3 ± 16.0
BSA (m^2^)	1.9 ± 0.2

Comorbidities, *n (%)*	
Hypertension	13 (32.5%)
Ischaemic heart disease	12 (30.0%)
Atrial fibrillation	8 (20.0%)
Heart failure	8 (20.0%)
Type 2 diabetes	4 (10.0%)
Hypercholesterolaemia	6 (15.0%)
COPD	3 (7.5%)
Smoking (never/former)	34 (85.0%)/6 (15.0%)
NYHA class	
Class I	31 (77.5%)
Class II	3 (7.5%)
Class III	4 (10.0%)
Class IV	2 (5.0%)

Laboratory findings	
Haemoglobin (g/L)	135.7 ± 16.4
NT-proBNP (pg/mL)	1175.3 ± 3616.9
Creatinine (µmol/L)	83.6 ± 18.3
Urea (mmol/L)	5.6 ± 1.8
eGFR (mL/min/1.73 m^2^)	72.0 ± 14.0

Data are given as mean ± standard deviation unless otherwise stated.

BSA, body surface area; COPD, chronic obstructive pulmonary disease; NYHA, New York Heart Association

**Inclusion criteria:** Age >18 years, clinical indication for CMR, and diagnostic quality 4D flow acquisition.

**Exclusion criteria:** body weight >120 kg, inability to lie flat, pregnancy, incompatible devices or implants or any other contraindication to CMR, including allergy to contrast, claustrophobia, and chronic kidney disease stage 4 or worse (estimated glomerular filtration rate <30 mL/min/1.73 m^2^).

### Ethics statement

The research adhered to the guidelines outlined in the Declaration of Helsinki. Data acquisition and handling were authorized by the National Research Ethics Service (reference: 21/NE/0149). A pragmatic opt-out informed consent was obtained from all subjects included in the study.

### CMR acquisition and analysis methods

CMR studies were performed on a 1.5 Tesla MAGNETOM Sola (Siemens Healthineers, Erlangen, Germany) system equipped with BioMatrix Body 18 coil technology. The CMR protocol comprised localizer/survey images, cine imaging, late gadolinium enhancement (LGE) imaging, and 4D-flow acquisition.

### Cine imaging

Cine images were acquired during end-expiratory breath-hold using a balanced steady-state free precession (bSSFP) sequence with retrospective ECG gating. Cine imaging included standard long-axis views (vertical long-axis, horizontal long-axis, three-chamber, and aortic root/LVOT views) and a contiguous short-axis left-ventricular stack acquired from base to apex.

For cines, 30 cardiac phases were reconstructed. Typical imaging parameters included an echo time (TE) of 1.13 ms, repetition time (TR) of 2.71 ms, flip angle of 55°, readout field of view (FOV) of 360 mm with a phase FOV of approximately 80% (resulting in an in-plane FOV of ∼360 × 289 mm^2^), and parallel imaging using GeneRalized Autocalibrating Partial Parallel Acquisition (GRAPPA) with an acceleration factor of 2. The acquisition matrix was 224 × 180, yielding an in-plane spatial resolution of approximately 1.6 × 1.6 mm^2^. Slice thickness was 8 mm with an inter-slice gap of 2 mm, and the number of slices was adjusted to ventricular size. Temporal resolution was heart-rate dependent.

### 4D-flow acquisition

Time-resolved three-dimensional, three-directional velocity-encoded phase-contrast MRI (4D flow) was acquired with retrospective ECG gating and reconstructed into 30 cardiac phases to ensure temporal consistency with cine imaging. The acquisition was performed during free breathing without respiratory navigator gating using a non-breath-hold protocol, with complete volumetric coverage of the thoracic aorta acquired in the axial plane. 4D flow was acquired following the administration of gadolinium-based contrast agent.

4D-flow imaging was performed using a spoiled gradient-echo–based velocity-encoded sequence with a flip angle of 15°. Echo time was approximately 2.7 ms for standard velocity-encoding acquisitions and was reduced for higher velocity-encoding settings. A segmented acquisition scheme was used, with an underlying gradient-echo repetition time on the order of 4–5 ms. Spatial resolution was approximately isotropic, with in-plane voxel dimensions of ∼3.1 × 3.1 mm^2^ and a slice thickness of 3.1 mm, acquired without inter-slice gap. In-plane FOV and phase FOV were adjusted to ensure full thoracic aortic coverage, resulting in an effective FOV of approximately 200 × 256 mm^2^, with minor variation according to patient size and protocol variant. Parallel imaging was applied using GRAPPA acceleration in the phase-encoding direction with an acceleration factor of 2 and no slice-direction acceleration. Temporal resolution was heart-rate dependent for the reconstructed 30 cardiac phases.

Velocity encoding (VENC) was set to 150 cm/s by default and increased up to 400 cm/s in the presence of stenotic valvular heart disease to mitigate velocity aliasing. Typical acquisition time for the free-breathing, non-navigator 4D-flow protocol was 8–12 min, depending on heart rate, spatial coverage, and breathing pattern.

### 4D-flow pre-processing and post-processing

Background phase-offset correction and related pre-processing steps were performed offline during post-processing using MASS software (Leiden University Medical Center). Quality control included visual inspection for residual phase offsets and velocity aliasing, with additional corrective steps applied as required prior to quantitative flow analysis.

### Stage 1: manual segmentation and validation

Image analysis was performed using the MASS research software (MASS, Version 2025-EXP, Leiden University Medical Center, Leiden, The Netherlands). Automated cine segmentation was performed, with manual review and correction if required.

The magnitude images from the 4D flow dataset were reconstructed into an axial stack, with a voxel size of 3 × 3 × 3 mm. Manual contours were drawn for all cardiac chambers (LV, RV, LA, and RA) and great vessels (Aorta and main pulmonary artery (MPA)). Three phases were segmented in the training set, all phases segmented in the test set. Papillary muscles and trabeculations were excluded in the blood pool volume calculation.^13^

For ventricular assessment, comparisons were made between the contoured magnitude images and values derived from the standard cine short-axis stack. Regarding the atria, as full cine stacks are not routinely contoured in all clinical workflows, 4D flow-derived volumes were compared against reported reference values using Z-scores.^14,15^

### Stage 2: AI model development

In stage 2, automated 3D segmentation of the LV, RV, LA, RA, aorta, and MPA was performed on the 4D flow MRI magnitude images using a 3D full-resolution nnU-Net model.^16^ The network was trained using manually segmented data from 30 subjects across three cardiac phases (end-diastole, end-systole, and mid-diastole), yielding a total of 90 image volumes. We applied the standard nnU-Net pipeline, which implicitly manages image resampling, intensity normalization, and hyper-parameter tuning. Training was conducted for 250 epochs on a workstation equipped with an NVIDIA GeForce GTX 1080Ti GPU (12 GB memory).

An initial five-fold cross-validation demonstrated consistently high DICE overlap scores (average 0.89 ± 0.004; range 0.84–0.94), confirming the adequacy of the default configuration and training set size. Consequently, the final model was trained on the complete 30-subject cohort to maximize performance. The network was then evaluated against an independent test set of 10 subjects with manual segmentations available for all cardiac phases. This independent testing yielded an average DICE score of 0.88, closely mirroring the cross-validation results and confirming the overall robustness of the segmentation network.

**Figure 1:**
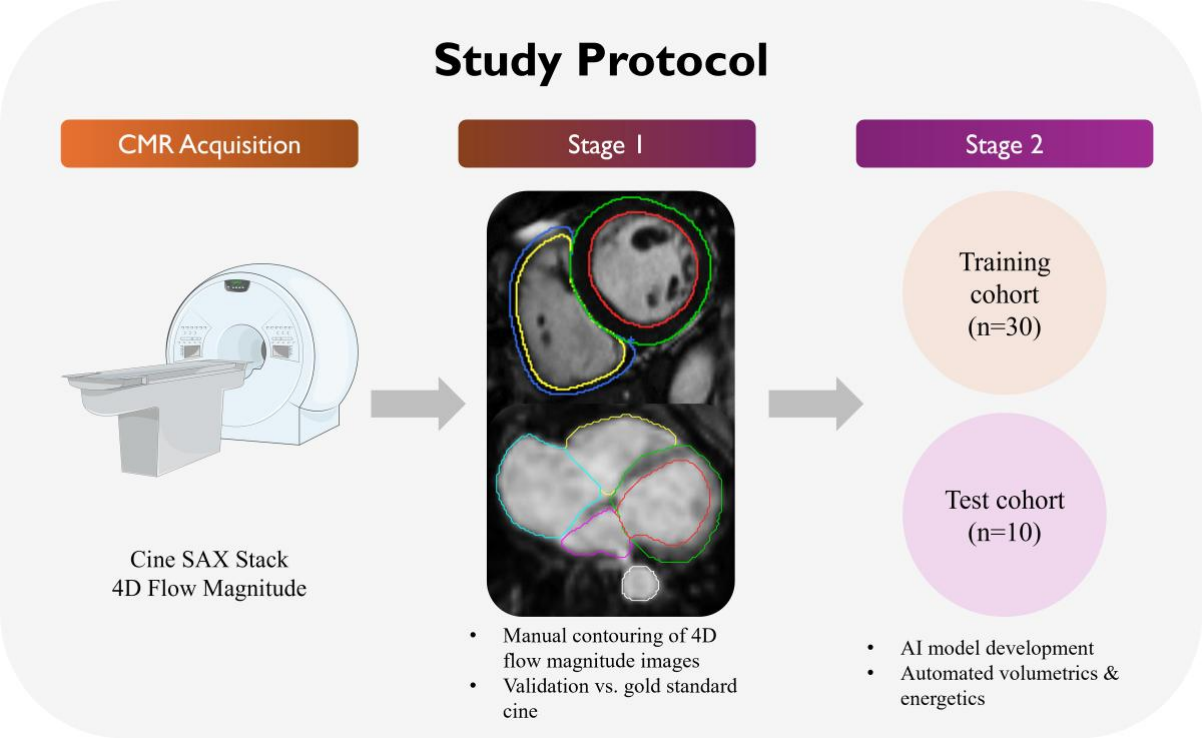
Overview of the study flow chart. CMR, cardiac magnetic resonance; SAX, short-axis.

An overview of the study flow chart is shown in *Figure 1*.

### Haemodynamic assessment

To demonstrate the functional utility of the AI segmentations, we computed advanced haemodynamic metrics. Kinetic energy (KE), vorticity, and viscous energy loss (EL) were calculated for all four cardiac chambers (LV, RV, LA, and RA) and the great vessels (aorta and MPA) over the cardiac cycle.^17,18^ These metrics were derived by integrating the 4D flow velocity field within the specific chamber masks generated by (i) manual contouring and (ii) the AI model. Comparisons were performed for the peak (maximum value over the cardiac cycle) metric.

To assess agreement for conventional flow metrics, standard aortic haemodynamic parameters were quantified from the 4D flow data in a representative subset of 10 cases using both the manual and the AI-derived aortic segmentations. Net stroke volume (SV), aortic forward flow (AFF), regurgitant volume (AR), and peak velocity (Vmax; the maximum velocity within the aortic region of interest over the cardiac cycle) were derived and compared between the two methods, with peak velocity reported in metres per second. Agreement was assessed using the same statistical approach as for the volumetric and energetic analyses.

### Statistical analysis

Normality was tested using the Shapiro–Wilk test, and visually inspected on histograms and Q–Q plots. Continuous variables are presented as mean ± standard deviation (SD) or median and interquartile range.

Correlations were assessed using Pearson’s correlation coefficient (*r*) for parametric data or Spearman coefficient of rank correlation (ρ) for non-parametric data. Intraclass correlation coefficient (ICC) was calculated and Bland-Altman analysis was used for bias and agreement. Paired *t*-tests or Wilcoxon signed-rank tests were used to assess for systematic differences between two methods. We calculated the within-subject coefficient of variation (CoV) as SD of the differences divided by the mean. Atrial volumes were assessed using Z-scores against normative means. The performance of the AI model was evaluated using the dice similarity coefficient (DSC), defined as the spatial overlap between the AI-generated contours and the manual ground truth contours divided by the average size of the two regions.

Intra-observer variability was assessed in a randomly selected subset of 10 patients by a single observer repeating the manual segmentation >4 weeks after the initial analysis to minimize recall bias. Inter-observer variability was assessed in a randomly selected subset of 5 patients by a second observer.

Statistical analyses were conducted using MedCalc (MedCalc Software, Ostend, Belgium). A *P*-value <0.05 was considered statistically significant.

## Results

### Baseline characteristics

The study cohort consisted of 40 patients. The clinical and demographic characteristics of the study population are summarized in *Table 1*. The mean age was 69.0 ± 17.2 years, and 24 (60.0%) were male. Comorbidities were prevalent, with 32.5% of patients having hypertension and 30.0% with ischaemic heart disease. Studies included a range of pathologies representative of a normal CMR workflow (*Table 2*).

**Table 2:** The pathology identified from the final clinical reports of the CMR scans included in the cohort

Pathology	All (*n* = 40)	Training (*n* = 30)	Validation (*n* = 10)
Normal, *n* (%)	12 (30)	11 (37)	1 (10)
Heart failure *n* (%)			
HFrEF	3 (8)	2 (7)	1 (10)
HFpEF	8 (20)	5 (17)	3 (30)
Valvular heart disease, *n* (%)	13 (33)	10 (33)	3 (30)
IHD, *n* (%)	5 (13)	4 (13)	1 (10)
Myocarditis, *n* (%)	2 (5)	2 (7)	0 (0)
Cardiomyopathy, *n* (%)	1 (3)	1 (3)	0 (0)
Post-valvular intervention, *n* (%)	3 (8)	2 (7)	1 (10)

Some cases demonstrated more than one pathology. Training and validation cohort used in developing automated pipeline in Stage 2 detailed further

HFrEF, heart failure with reduced ejection fraction; HFpEF, heart failure with preserved ejection fraction; IHD, ischaemic heart disease

### Stage 1: manual 4D flow vs. standard cine comparators

#### Ventricular volumes

**Figure 2:**
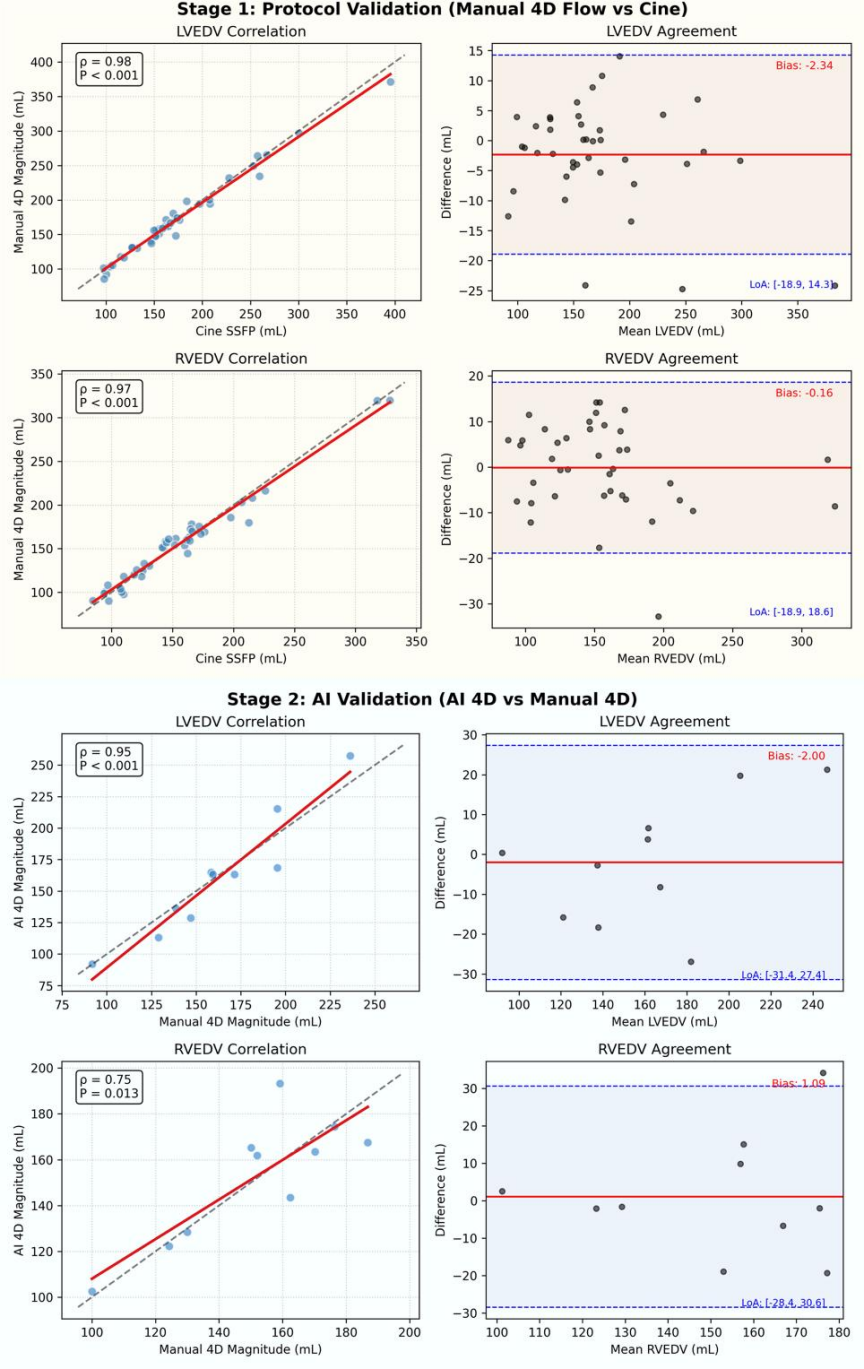
Validation of 4D flow magnitude volumetrics. **Top Panel (Stage 1): Protocol Validation.** Comparison of volumetric measurements derived from manual segmentation of 4D flow magnitude images against the clinical gold-standard cine SSFP. Strong correlations (ρ = 0.97) confirm that 4D flow magnitude images provide anatomical accuracy comparable to standard cine. **Bottom Panel (Stage 2): AI Validation.** Comparison of the automated deep learning (AI) model against manual segmentation of 4D flow magnitude images in the validation cohort. **Left Column:** Scatter plots demonstrating correlation between methods. The solid red line represents the linear regression, while the dashed black line indicates the line of identity (y = x). Spearman’s correlation coefficients (ρ) and *P*-values are displayed. **Right Column:** Bland-Altman plots assessing agreement. The solid red line indicates the mean bias (difference), and the dashed blue lines represent the 95% Limits of Agreement (±1.96 SD). LVEDV, left ventricular end-diastolic volume; RVEDV, right ventricular end-diastolic volume; LoA, limits of agreement.

4D flow magnitude analysis of the left ventricle showed excellent agreement with cine SSFP (*Figure 2*, *Table 3*). Median (IQR) left ventricular end-diastolic volume (LVEDV) derived from cine was 159.9 mL (130.35–190.61) compared to 158.3 mL (130.80–194.37) from 4D flow, with a mean bias of −2.3 mL (*P* = 0.20). LV end systolic volume (ESV) showed a minimal mean bias of +0.2 mL (65.8 (43.66–84.11) vs. 69.6 (42.05–84.09) mL, *P* = 0.79). LV stroke volume (SV) measured 95.2 (81.77–118.34) mL on cine and 90.7 (80.64–113.66) mL on 4D flow (mean bias −2.3 mL, *P* = 0.07). LV ejection fraction (EF) assessment showed excellent concordance 61.1 (57.05–67.31) vs. 59.6 (53.20–68.33) (mean bias +0.1%, *P* = 0.87). All parameters demonstrated Spearman correlation and ICC of ≥0.92.

**Table 3:** Stage 1 results

	Correlation (ρ)	ICC	*P*-value
Left ventricle			
EDV (mL)	0.98	0.99	<0.05
ESV (mL)	0.97	0.98	<0.05
SV (mL)	0.95	0.96	<0.05
EF (%)	0.90	0.92	<0.05
Right ventricle			
EDV (mL)	0.97	0.98	<0.05
ESV (mL)	0.99	0.99	<0.05
SV (mL)	0.94	0.94	<0.05
EF (%)	0.90	0.87	<0.05

Comparison of ventricular volumetrics: 4D flow magnitude vs. standard cine.

Correlations are Spearman rank correlation coefficient (ρ). *P*-value for correlation.

Analysis of the right ventricle also demonstrated strong agreement. Comparison of mean right ventricular end-diastolic volume was 155.1 ± 52.8 mL for cine vs. 155.0 ± 50.4 mL for 4D flow (bias −0.1 mL, *P* = 0.92). Small but statistically significant biases were noted for RVESV (65.3 ± 28.0 vs. 68.0 ± 29.1 mL, +2.4 mL, *P* < 0.05) and accordingly the RVSV (89.9 ± 29.7 vs. 85.6 ± 25.6 mL, −4.3 mL, *P* < 0.05). Spearman correlation was ≥ 0.90 for all parameters and ICC was ≥ 0.87.

#### Atrial volumes

Atrial volumes derived from 4D flow fell within expected normative ranges. For the left atrium, mean LA ESV was 104.9 ± 45.3 mL (Z-score 0.24 ± 1.79, *P* = 0.40), and mean LA EDV was 89.5 ± 64.4 mL (Z-score 0.98, *P* = 0.11). For the right atrium, mean RA ESV was 115.9 ± 48.1 mL (Z-score 0.49 ± 1.59, *P* = 0.06), and mean RA EDV was 89.3 ± 61.7 mL (Z-score 0.97, *P* < 0.05; see Supplementary data online, *Table S1*).

### Stage 2: AI model performance and haemodynamic validation

**Figure 3:**
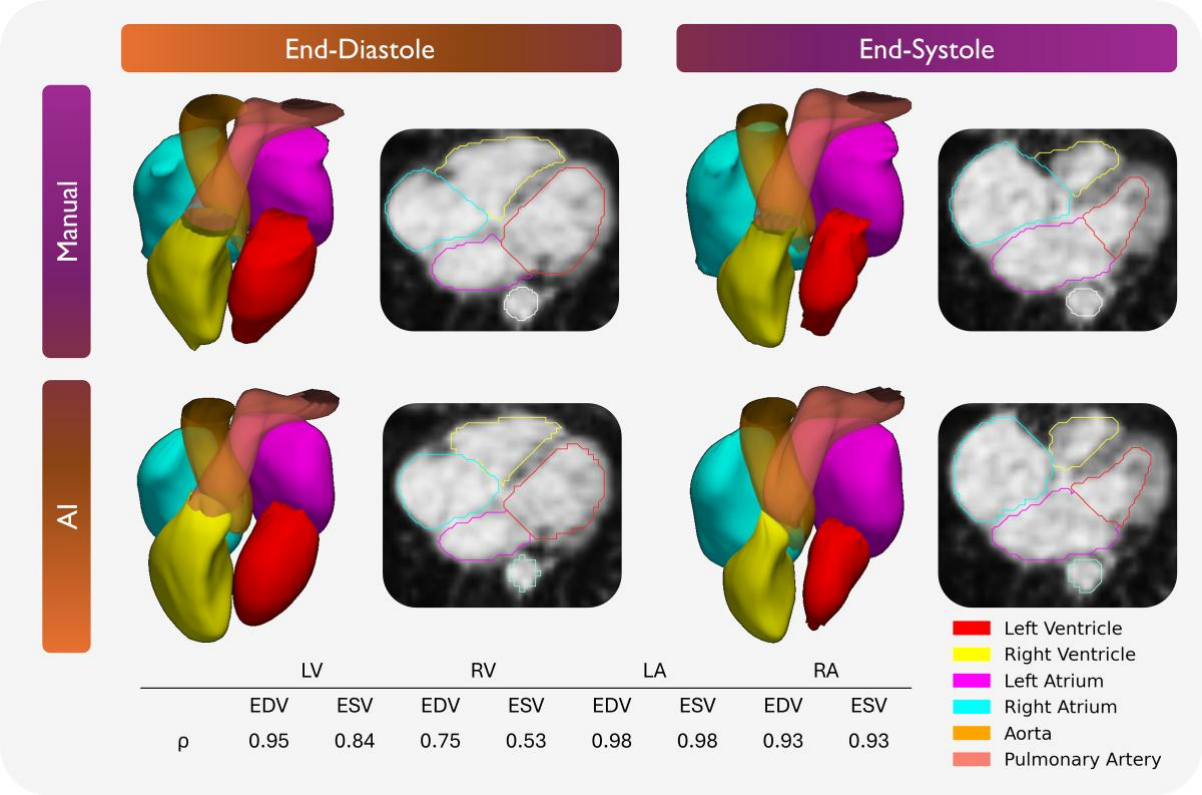
Visualisation of automated 4D flow magnitude segmentation. Representative case from the validation cohort demonstrating the performance of the deep learning model. **(Top & Bottom Panels)** 3D surface reconstructions of the cardiac chambers and great vessels derived from Manual (left column) and AI (right column) segmentation at end-diastole and end-systole. **(Middle Panel)** Corresponding 2D axial slices from the 4D flow magnitude stack overlaid with segmentation contours, showing precise boundary definition. **Colour Key:** Left ventricle (red); right ventricle (yellow); left atrium (pink); right atrium (blue); ascending aorta (orange [3D reconstruction]); pulmonary artery (purple). EDV, end-diastolic volume; ESV, end-systolic volume; LV, left ventricle; RV, right ventricle; LA, left atrium; RA, right atrium. ρ = correlation for manual vs. AI segmentation, given as Spearman rank correlation coefficient.

The deep learning model successfully segmented cardiac chambers with an overall mean DSC of 0.88. Segmentation performance was highest for the aorta (DSC 0.92), followed by the left atrium and right atrium (both DSC 0.89) (*Figure 3*).

Comparisons of volumetrics between manual and AI-derived contours revealed mixed results (*Figure 2*, *Table 4*). For the LV, agreement was strong for LVEDV (ICC 0.94), though a bias in LVESV (−8.8 mL, *P* < 0.05) contributed to an overestimation of LVEF (+4.6%, *P* < 0.05). RV volumetrics showed generally strong agreement (ICC ≥0.74 for all metrics) without significant difference in mean values.

**Table 4:** Stage 2 results. comparison of volumetrics: manual vs. AI segmentation, intra- and inter-observer variability results

Parameter	Manual vs. AI (*n* = 10)		Intra-observer (*n* = 10)		Inter-observer (*n* = 5)	
	ρ	CoV (%)	ρ	CoV (%)	ρ	CoV (%)
Left ventricle						
EDV (mL)	0.95*	6.3	0.86*	6.0	0.90*	12.8
ESV (mL)	0.84*	13.3	0.83*	7.1	0.70	26.2
SV (mL)	0.93*	6.5	0.81*	15.1	0.90*	17.1
EF (%)	0.78*	6.3	0.89*	11.0	0.80	18.3
Right ventricle						
EDV (mL)	0.75*	6.3	0.87*	7.1	0.70	8.7
ESV (mL)	0.53	9.9	0.70*	23.2	0.90*	7.5
SV (mL)	0.90*	8.4	0.83*	9.2	0.40	18.1
EF (%)	0.82*	6.5	0.86*	13.0	0.70	11.0
Left atrium						
EDV (mL)	0.98*	9.6	0.90*	9.8	0.70	15.7
ESV (mL)	0.98*	6.7	0.73*	10.9	1.00*	19.1
SV (mL)	0.76*	24.2	0.56	28.1	0.10	42.7
EF (%)	0.90*	20.4	0.84*	23.6	0.30	33.5
Right atrium						
EDV (mL)	0.93*	9.9	0.92*	18.8	0.90*	10.8
ESV (mL)	0.93*	6.4	0.95*	6.9	0.60	11.8
SV (mL)	0.78*	18.8	0.90*	15.4	0.90*	40.2
EF (%)	0.96*	15.5	0.97*	12.8	0.90*	30.0

Correlation given as Spearman rank correlation coefficient (ρ).

EDV, end diastolic volume; ESV, end systolic volume; SV, stroke volume; EF, ejection fraction; CoV, coefficient of variance

^*^indicates *P* < 0.05.

For the atria, the AI model showed excellent correlation for left atrial ESV (ρ = 0.98, ICC = 0.96) and right atrial ESV (ρ = 0.93, ICC = 0.95) (*Table 4*), although a systematic bias was observed with the AI model underestimating atrial ESV (LA bias −11.0 mL, *P* < 0.05; RA bias −10.5 mL, *P* < 0.05).

#### Energetics

**Figure 4:**
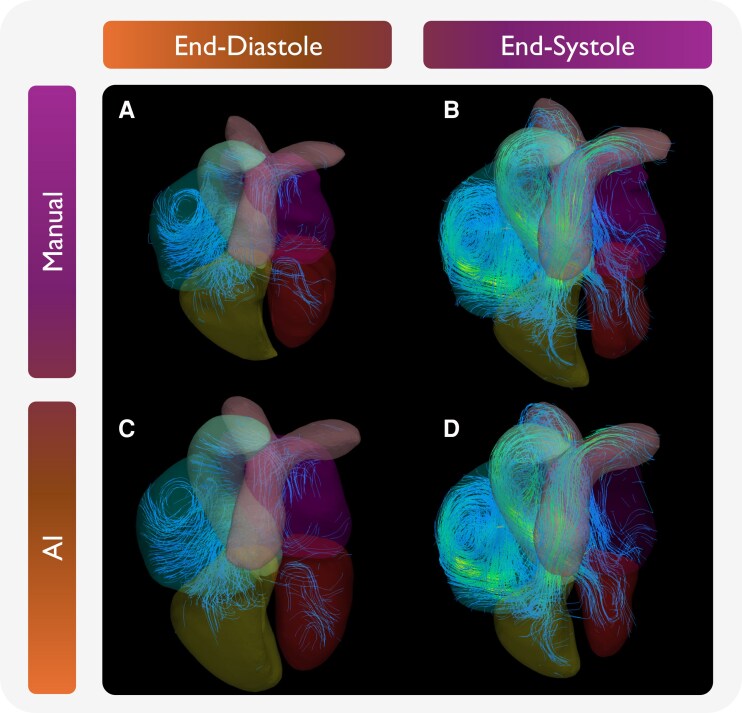
Visualization of intracardiac flow streamlines. Representative whole-heart 3D flow streamlines derived from manual and automated (AI) segmentations. The 4D flow velocity field is integrated within the respective chamber boundaries to visualize complex haemodynamics. (*A* & *B*) Streamlines generated using manual ground-truth segmentations at end-diastole (*A*) and end-systole (*B*). (*C* & *D*) Corresponding streamlines generated using the fully automated AI segmentations at end-diastole (*C*) and end-systole (*D*). The strong qualitative visual agreement between the manual and AI-derived streamlines demonstrates the AI model's reliability in accurately masking the 4D flow velocity data for whole-heart physiological assessment. **Colour Key:** Left ventricle (red); right ventricle (yellow); left atrium (pink); right atrium (blue); aorta (orange); pulmonary artery (purple).

Comparison of advanced haemodynamic indices derived from manual vs. AI contours demonstrated generally strong agreement, particularly for correlations (*r* > 0.88 KE, Vo, EL across all four chambers) (*Table 5*, *Figure 4*).

**Table 5:** Manual vs. automated energetics analysis

Variable	Correlation (r)	*P*-value	CoV (%)
Left ventricle			
KE (µJ/mL)	0.96	<0.05	9.8
Vo (s^−1^)	0.89	<0.05	8.9
EL (µW)	0.98	<0.05	12.4
Right ventricle			
KE (µJ/mL)	0.88	<0.05	11.6
Vo (s^−1^)	0.91	<0.05	6.5
EL (µW)	0.95	<0.05	27.0
Left atrium			
KE (µJ/mL)	0.92	<0.05	11.9
Vo (s^−1^)	0.99	<0.05	5.3
EL (µW)	0.96	<0.05	28.0
Right atrium			
KE (µJ/mL)	0.98	<0.05	11.4
Vo (s^−1^)	0.99	<0.05	3.2
EL (µW)	0.91	<0.05	15.8

Correlation given as Pearson correlation coefficient (r). *P*-value for correlation.

KE, kinetic energy; Vo, vorticity; EL, energy loss; CoV, coefficient of variance.

The left ventricle demonstrated the most consistent results, with no statistically significant bias between methods and an ICC of ≥0.87 for all metrics. The right ventricle showed a statistically significant difference in mean EL (1406 ± 760.3 µW for manual vs. 985 ± 529.8 µW for AI, *P* < 0.05) with a CoV of 27.0%. However, the CoV for the interobserver variability analysis was 39.6% for RV EL.

Left atrial energetics demonstrated significant bias for all parameters (KE −1.1 µJ/mL, Vo −15.7 s^−1^, EL −306.6 µW). CoV for these parameters compared to interobserver variability analysis was favourable (11.9% vs. 56.5%, 5.3% vs. 15.3% and 28.0% vs. 42.5% respectively). Right atrial energetics demonstrated better concordance, with a significant difference demonstrated only for EL (bias −177.6 µW, *P* < 0.05). CoV vs. interobserver was 15.8% vs. 43.3%, respectively.

#### Standard aortic flow

Agreement between manual and AI-derived standard aortic flow parameters was strong (*Table 6*). Stroke volume and aortic forward flow demonstrated excellent agreement (*r* = 0.98, ICC ≥ 0.96 for both). Peak velocity also agreed well (*r* = 0.92, ICC = 0.93). Aortic regurgitant volume showed more modest agreement (*r* = 0.77, ICC = 0.75), likely reflecting the small regurgitant volumes in this predominantly non-regurgitant cohort and the greater sensitivity of small-volume regurgitation to segmentation boundaries.

**Table 6:** Manual vs. automated standard aortic flow analysis

Variable	Correlation (*r*)	ICC	*P*-value
SV (mL)	0.98	0.97	<0.05
AFF (mL)	0.98	0.96	<0.05
AR (mL)	0.77	0.75	<0.05
Vmax (m/s)	0.92	0.93	<0.05

SV, stroke volume; AFF, aortic forward flow; AR, aortic regurgitant volume; *V*_max_, peak velocity (maximum velocity within the aortic region of interest over the cardiac cycle, reported in metres per second). ICC, intraclass correlation coefficient. Correlation given as Pearson correlation coefficient (*r*). *P*-value for correlation. *n* = 10.

### Intra- and inter-observer variability

Manual segmentation of 4D flow magnitude images demonstrated excellent intra-observer reproducibility (*n* = 10), with negligible bias and ICC ≥0.98 for all ventricular volumetrics. Left atrial volumes showed negligible bias with ICC ≥0.87, while right atrial ESV demonstrated a significant bias of −10.45 mL with ICC ≥0.77 (*Table 4*). Inter-observer analysis (*n* = 5) revealed more heterogeneity. There was significant bias for LVESV (−30.13 mL), LAESV (+23.95 mL) and RAESV (+21.65 mL) with agreement for volumetric measures ranging from ICC 0.47–0.87. Correlation between observers for energetics analysis was generally good, with ICC (observer 1 vs. 2 vs. AI) for KE of 0.83–0.96, Vo 0.82–0.98 and EL 0.76–0.96 across cardiac chambers (see Supplementary data online, *Table S2*).

## Discussion

In this study, we developed and validated a workflow for deriving cardiac volumetrics and advanced flow energetics directly from 4D flow magnitude images. We demonstrated that (i) manual segmentation of 4D flow magnitude images yields ventricular volumes comparable to gold-standard short-axis cines with high correlation (*r* > 0.96 for all volumes) and (ii) a deep learning model can automate this process with high Dice similarity scores (>0.85 for all chambers).

### Anatomical accuracy of 4D flow magnitude

Our results confirm that 4D flow magnitude images are a reliable source for volumetric assessment. The correlation with cine data was excellent (ICC > 0.96 for all volumes). These findings align with recent work by Reiter *et al*.^10^ who also demonstrated high correlations between native 4D flow magnitude and cine volumetrics (*r* > 0.95). Interestingly, Reiter *et al*.^10^ reported a slight underestimation of LV volumes (−2.9 mL EDV) using 4D flow magnitude images, comparable to the bias seen in our study (−2.3 mL), with both biases being small and likely clinically insignificant. The atrial Z-scores confirmed that 4D flow magnitude images capture atrial geometry consistent with normative data, validating their use in the absence of dedicated atrial cine stacks.

### Variability and reproducibility

The variability observed in our study mirrors trends reported in the wider literature, where reproducibility is strongly influenced by observer experience and the imaging method used. Our intra-observer variability was excellent (ICC >0.98), consistent with values reported for experienced observers in standard cine analysis.^19^ However, our inter-observer variability was more heterogeneous, particularly for RV and functional parameters. While some studies report high inter-observer reproducibility for expert readers on standard cine (ICC >0.90),^20^ our results align more closely with findings for less experienced observers or more complex anatomies, where RV reproducibility has been reported to be significantly lower (ICC >0.46).^19^ This discrepancy likely reflects the inherent challenge of performing manual segmentation on 4D flow magnitude images, which typically have lower spatial resolution and contrast-to-noise ratios compared to optimized bSSFP cine sequences.

Regarding atrial volumetrics, previous studies using dedicated cine protocols have reported good reproducibility (ICC >0.88).^21,22^ Our study showed lower inter-observer agreement for atrial parameters, again likely due to the difficulty of delineating atrial walls in the context of lower spatial resolution seen with 4D flow magnitude images. Crucially, the introduction of our AI model addresses this issue of variability. As demonstrated by Bhuva *et al*.^11^ automated approaches provide deterministic consistency (zero intra-algorithm variability) and have been shown to achieve precision comparable to expert human analysis. By removing reader-dependent error, our AI pipeline offers a standardized, reproducible method for extracting these metrics from 4D flow data.

### Learning curve and technical challenges

A critical factor influencing manual analysis is the significant learning curve associated with interpreting 4D flow magnitude images. Unlike standard 2D cine stacks, which are the cornerstone of routine clinical CMR training, volumetric segmentation of 4D flow magnitude images is not a standard task. The lower spatial resolution and distinct tissue contrast of magnitude images require specific acclimatization and dedicated training to achieve high reproducibility. Furthermore, this manual process is exceptionally labour-intensive; comprehensive segmentation of all cardiac phases can take up to four hours per case. In contrast, the proposed AI pipeline completes this task in approximately two minutes. This highlights a key advantage of automation: it eliminates the observer dependence and potential variability associated with the steep learning curve of interpreting low-contrast magnitude images, while simultaneously resolving the prohibitive time burden inherent in this method of post-processing.

### AI automation performance and energetic assessment

The AI model demonstrated robust performance across all cardiac chambers and great vessels, with particularly high accuracy in the aorta (DSC 0.92) and atria (DSC 0.89). Although LV segmentation exhibited a systematic bias in ESV (−8.8 mL), resulting in a slight overestimation of EF by 4.6%, the strong correlations indicate that this offset is predictable and potentially correctable. Crucially, the automation offered by this model is the key enabler for a ‘One-Stop Shop’ protocol. By rapidly deriving four-chamber volumes and function, and great vessel dimensions from the magnitude data, and combining this with the haemodynamic information inherent to the 4D flow dataset, clinicians can obtain a comprehensive whole-heart physiological assessment from a single free-breathing acquisition. The strong agreement in derived energetics (KE, vorticity, energy loss), particularly in the left ventricle, further validates that the AI contours are not only anatomically reasonable, but also functionally sufficient for advanced haemodynamic analysis. Agreement extended to conventional aortic flow metrics, with stroke volume and forward flow showing excellent concordance between manual and automated contours, supporting the applicability of the pipeline to routine clinical flow quantification.

### Limitations

This study has several limitations. This was a single-centre study with a relatively small cohort of 40 patients. While the AI model achieved good Dice scores, the systematic bias observed in LVESV suggests that further training with a larger, more diverse dataset is required to improve accuracy in systolic phases. All data were acquired on a single 1.5 T scanner from one vendor; external validation across scanner vendors and field strengths is a necessary next step.

Regarding the haemodynamic results, while correlations between AI and manual derived metrics were strong, significant biases were observed, particularly for right ventricular energy loss (−421.3 µW) and atrial parameters. These discrepancies are likely attributable to small differences in segmentation boundaries. With energy loss primarily taking place near the chamber wall, there is increased susceptibility to deviations in energy loss calculation driven by small alterations in segmentation.

## Conclusion

4D flow CMR magnitude images allow accurate four chamber volumetric analysis. When coupled with deep learning automation, this approach facilitates a rapid, comprehensive assessment of cardiac structure, function, and advanced flow energetics from a single free-breathing acquisition.

## Supplementary Material

qyag125_Supplementary_Data

## Data Availability

The data underlying this article will be shared on reasonable request to the corresponding author.

## Lead author biography

Dr Alexander Gall is an Advanced Cardiac Imaging Research Fellow at the University of East Anglia and a Cardiology Registrar in Norwich, UK. He has a strong clinical focus on multimodality imaging. His research is dedicated to the clinical translation of 4D flow CMR, specifically focusing on right heart flow haemodynamics, advanced energetics, and the automation of imaging workflows.
