# Supplementary material for: Automated whole-heart volumetrics and haemodynamics from 4D flow CMR magnitude images: development and validation of a deep learning model
Source: Eur Heart J Imaging Methods Pract. 2026 Jul 9;4(3):qyag125. doi: 10.1093/ehjimp/qyag125 (PMC13398991; doi:10.1093/ehjimp/qyag125)

#### Supplementary Table 1: Mean atrial volume vs mean Z score from published values.

|  | **Mean Volume** | **Mean Z Score** | **p-value** |
| --- | --- | --- | --- |
| Left Atrium |  |  |  |
| ESV | 104.9 ± 45.29 | 0.24 ± 1.79 | 0.40 |
| EF | 43.5 ± 15.42 | -0.55 ± 1.98 | 0.08 |
| Right Atrium |  |  |  |
| ESV | 115.9 ± 48.07 | 0.49 ± 1.59 | 0.06 |
| EF | 46.8 ± 13.50 | -0.05 ± 1.63 | 0.84 |

ESV = end systolic volume. EF = ejection fraction.

#### Supplementary Table 2: Interobserver variability analysis of energetics.

| Variable | Correlation (r) | *p*-value | CoV (%) |
| --- | --- | --- | --- |
| Left Ventricle |  |  |  |
| KE (µJ/mL) | 0.63 | 0.25 | 44.3 |
| Vo (s^-1^) | 0.68 | 0.20 | 28.7 |
| EL (µW) | 0.83 | 0.08 | 28.3 |
| Right Ventricle |  |  |  |
| KE (µJ/mL) | 0.70 | 0.19 | 42.7 |
| Vo (s^-1^) | 0.98 | <0.05 | 22.5 |
| EL (µW) | 0.93 | <0.05 | 39.6 |
| Left Atrium |  |  |  |
| KE (µJ/mL) | 0.50 | 0.39 | 56.5 |
| Vo (s^-1^) | 0.99 | <0.05 | 15.3 |
| EL (µW) | 0.81 | 0.10 | 42.5 |
| Right Atrium |  |  |  |
| KE (µJ/mL) | 0.86 | 0.06 | 32.8 |
| Vo (s^-1^) | 0.99 | <0.05 | 12.8 |
| EL (µW) | 0.48 | 0.42 | 43.3 |

KE = kinetic energy. Vo = vorticity. EL = energy loss. CoV = coefficient of variance.

Correlation given as Pearson correlation coefficient (r). p-value for correlation.

#### Supplementary Figure 1: Validation of Advanced Haemodynamic Energetics.

Agreement between manual and AI-derived segmentation for advanced flow parameters across all four cardiac chambers. **Columns:** Comparison of Peak Kinetic Energy (Left), Peak Vorticity (Middle), and Peak Viscous Energy Loss (Right). **Rows:** Left Ventricle (LV), Right Ventricle (RV), Left Atrium (LA), and Right Atrium (RA). Scatter plots display individual data points (n=10) with the line of identity (dashed black) and linear regression (solid red). Pearson correlation coefficients (r) and p-values are reported for each parameter. The AI model demonstrates strong correlations (r > 0.88) for all metrics, indicating functional equivalence to manual analysis.


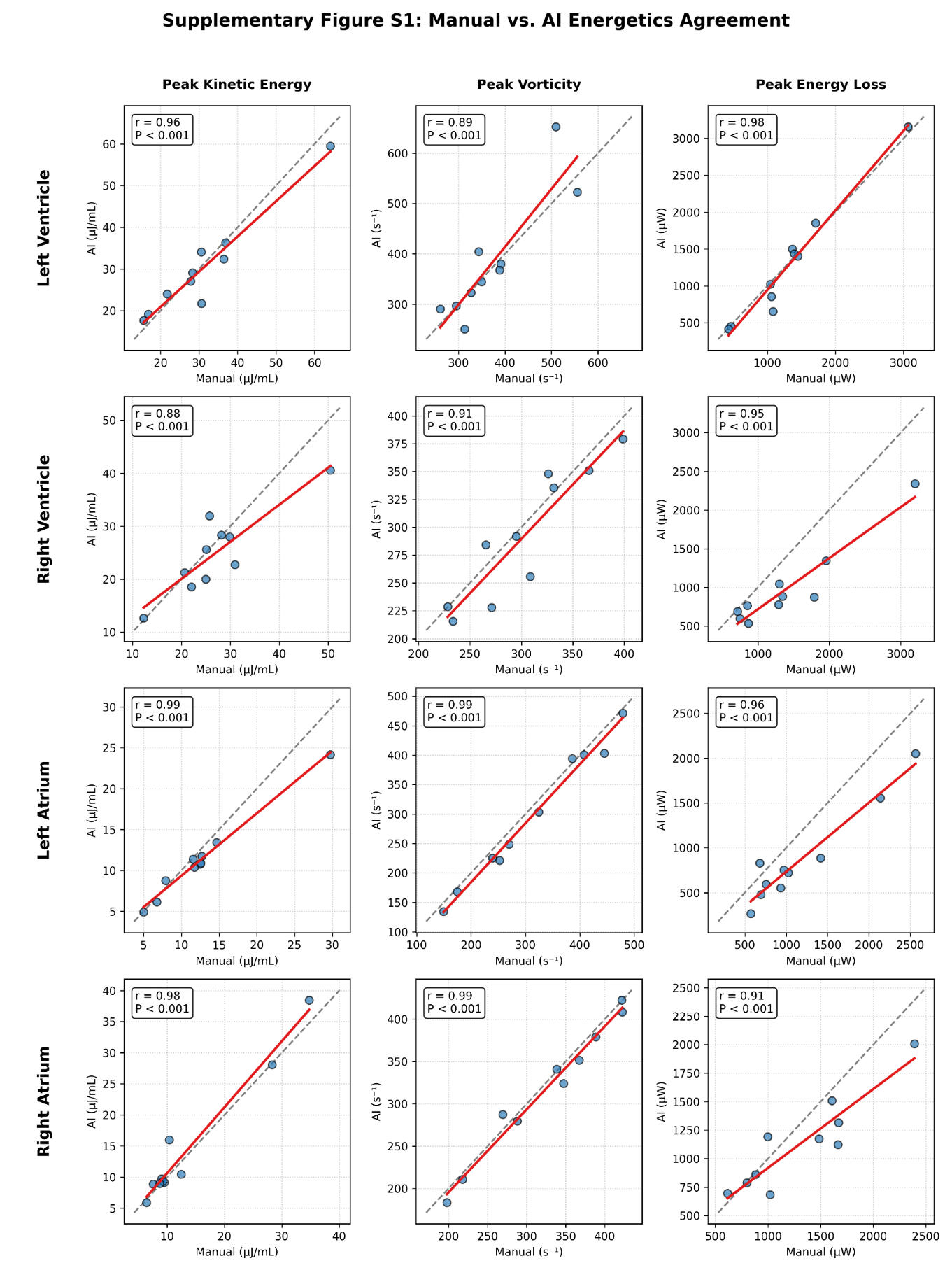

Supplement: qyag125_Supplementary_Data [file qyag125_supplementary_data.docx]
